# Rv1899c, an HDAC1–ZBTB25-Interacting Protein of *Mycobacterium tuberculosis*, Promotes Stress Resistance and Immune Evasion in Infected Macrophages

**DOI:** 10.3390/ijms262210872

**Published:** 2025-11-09

**Authors:** Arjun M. Menon, Boinapalli Gopichand, Shwetha Susan Thomas, Kuniyil Abhinand, Bipin G. Nair, Geetha B. Kumar, Pradeesh Babu, KB Arun, Lekshmi K. Edison, Aravind Madhavan

**Affiliations:** 1 School of Biotechnology, Amrita Vishwa Vidyapeetham, Amritapuri, Kollam 690525, Kerala, India; 2 Department of Life Sciences, CHRIST (Deemed to be University), Bangalore 560029, Karnataka, India; 3 Department of Comparative, Diagnostic, and Population Medicine, College of Veterinary Medicine, University of Florida, Gainesville, FL 32608, USA

**Keywords:** immune evasion, epigenetics, macrophage polarization, tuberculosis, autophagy

## Abstract

Rv1899c, a previously identified HDAC1–ZBTB25-interacting protein of *Mycobacterium tuberculosis*, plays a crucial role in bacterial adaptation and immune modulation. Recombinant *M. smegmatis*-expressing *Rv1899c* (MS_ *Rv1899c*) showed enhanced survival under acidic and oxidative stress compared to vector controls, along with improved early intracellular growth in THP1-derived macrophages. This was accompanied by reduced reactive oxygen species (ROS), diminished cytokines associated with inflammation and downregulation of autophagy proteins ATG5, Beclin, and LC3, which ultimately skewed the immune response, suppressing the pro-inflammatory M1 macrophage population. Targeting Rv1899c with 3-aminobenzamide (3-AB) impaired intracellular bacterial survival and restored IL-12B expression, while its combination with the HDAC inhibitor C1994 significantly enhanced bacterial clearance. Structural modelling confirmed the high stereochemical quality of the Rv1899c macrodomain, and computational studies identified 3-AB as the strongest ligand (−5.75 kcal/mol), stabilized through hydrogen bonding and hydrophobic interactions with key residues. Molecular dynamics simulations conducted for 200 ns demonstrated stable protein–ligand interactions with consistent parameters, while MM/GBSA analysis indicated favourable binding energy (ΔG_bind = −6.6 kcal/mol), largely influenced by van der Waals and electrostatic forces. Together, these findings highlight *Rv1899c* as a mediator of stress resistance and immune evasion and propose it as a potential therapeutic target against *M. tuberculosis*.

## 1. Introduction

The bacterial disease tuberculosis (TB) caused by *Mycobacterium tuberculosis* (*M. tuberculosis*) continues to rank as a major infectious illness globally. Based on the World Health Organization’s report, in the year 2023, approximately 10.6 million people developed TB, resulting in 1.3 million deaths worldwide; India contributed significantly to these cases. This represents nearly one in four of the global TB cases and deaths [1]. The development and propagation of multidrug-resistant and extensively drug-resistant strains undermines a serious challenge to global TB control, compromising the effectiveness of first- and second-line therapies. Therapy for drug-resistant tuberculosis requires prolonged use of multiple antibiotics, a strategy that is often complicated by drug-related toxicities, limited patient compliance, and substantial relapse rates [2]. The obstacles associated with the current regimens reinforce the critical importance of developing novel interventions that go beyond standard antibiotic discovery, encompassing host-directed therapies and approaches targeting bacterial virulence factors.

Through sophisticated immune-evasion strategies, *M. tuberculosis* has adapted to persist inside host macrophages, ensuring its long-term survival [3]. This persistence is largely facilitated by *M. tuberculosis* manipulation of host signalling pathways, its suppression of pro-inflammatory mediators, interference with autophagy, and promotion of alternatively activated macrophage phenotypes. A number of *M. tuberculosis* genome-encoded proteins (‘Rv’ proteins) have been implicated in immune modulation [4,5,6]. ESAT-6 promotes phagosomal disruption and autophagy inhibition, Eis acetylates host targets to attenuate cytokines and autophagic responses, and PtpA interferes with phagosome acidification by blocking vacuolar H^+^-ATPase recruitment [7,8,9,10,11]. Such evidence highlights the critical need to investigate individual *M. tuberculosis* effectors, as their distinct contributions to immune modulation are central to the pathogen’s capacity for persistence.

Our previous study [12] identified an epigenetic repressor complex involving HDAC1 and ZBTB25 that is recruited during *M. tuberculosis* infection to silence IL-12B expression in macrophages. Inhibiting HDAC1 and ZBTB25 restored IL-12B production, stimulated autophagic clearance, and reduced intracellular bacterial survival, thereby revealing how *M. tuberculosis* manipulates host epigenetic programmes to evade immunity. These findings also provide a rationale for host-directed therapeutic strategies. Building upon this, we investigated Rv1899c, an ADP-ribosyl polymerase, which primarily interacts with HDAC1 and was postulated to exert a direct role in suppressing immune activity.

The present study delineates the role of *Rv1899c* in modulating host immune responses during mycobacterial infection. We demonstrate that *Rv1899c* exerts multifaceted effects: suppressing cytokines that facilitate inflammation (IL-6, IL-1β, IL-12B), impairing autophagy by downregulating ATG5, Beclin, and LC3, attenuating ROS production and promoting macrophage polarization toward non-bactericidal states. These combined actions create an intracellular environment that supports bacterial persistence. Functional inhibition with 3-aminobenzamide, a broad-spectrum ADP-ribosyl polymerase inhibitor, establishes that the enzymatic activity of Rv1899c is indispensable for these functions, as inhibition restores host immune responses and restricts bacterial survival.

## 2. Results

### 2.1. Structural Validation of the Rv1899c a Macrodomain-Containing Protein

**Figure 1:**
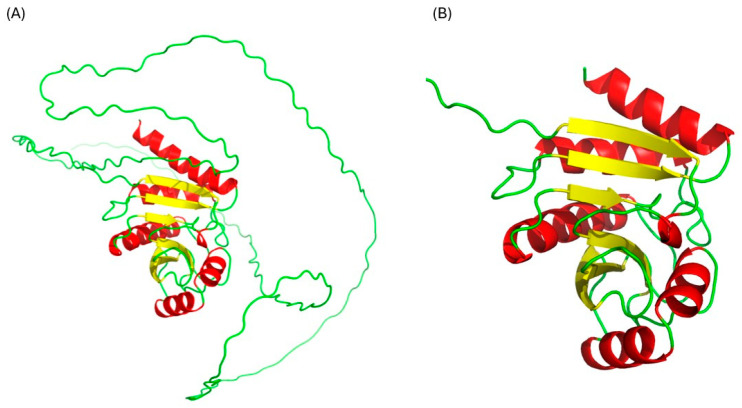
Structure of uncharacterized protein Rv1899c. (**A**) Complete structure, (**B**) macrodomain.

**Figure 2:**
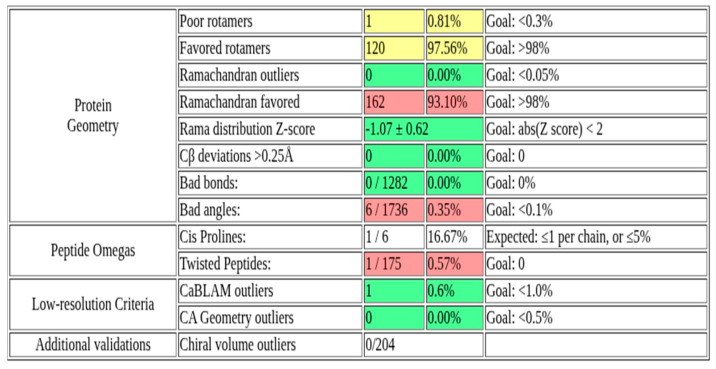
MolProbity validation summary of Rv1899c macrodomain 3D structure.

The three-dimensional structure of Rv1899c macrodomain obtained from AlphaFold (Figure 1) protein structure database was meticulously assessed to confirm the structural integrity and stereochemical quality. Figure 2 presents the validation summary of the macrodomain 3D structure obtained from MolProbity server. This analysis shows that 93.1% (162 out of 174) of the residues are in the most favoured regions and 100% are in allowed regions, with no outliers. These findings were further supported by the PROCHECK analysis, as elucidated in the Ramachandran plot. The Ramachandran plot shows 89.2% of the residues are present in the most favoured regions, and the remaining 10.8% in additionally allowed regions, with no residues in the disallowed regions. The analysis of side-chain geometry by the MolProbity server further confirmed the high quality of 3D structure beyond the backbone torsion angles. This analysis revealed that 97.56% of rotamers are present in favoured conformations, while other geometric parameters such as covalent bond quality and cB deviations are within the acceptable ranges. Overall, this quality assessment confirms that the 3D structure is of high quality and is suitable for the subsequent molecular docking and simulation studies.

### 2.2. Rv1899c Expression Enhances the Intracellular Multiplication of Recombinant M. smegmatis

**Figure 3:**
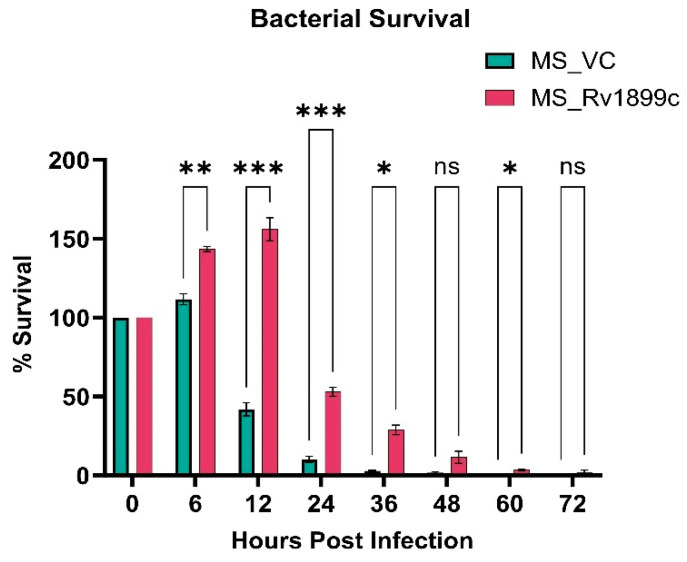
*Rv1899c* enhances bacterial survival within macrophages. THP-1-derived macrophages were infected with recombinant *M. smegmatis*-expressing *Rv1899c* (MS_*Rv1899c*) or vector control (MS_VC). Intracellular bacterial survival was assessed at the indicated time points post-infection by plating lysed macrophages on 7H10 agar and determining CFUs. Data are represented as percentage survival relative to the 0 h time point. Values represent mean ± SD from three independent experiments. Statistical significance was determined using one-way ANOVA followed by Šidák’s post hoc test. *p* ≤ 0.05 (*), *p* ≤ 0.01 (**), *p* ≤ 0.001 (***), ns: not significant.

The *Rv1899c* gene was amplified from *M. tuberculosis* H37Rv genomic DNA and cloned into the constitutive mycobacterial expression vector pBEN. The recombinant plasmid was introduced into *M. smegmatis* by electroporation, and *M. smegmatis* harbouring the empty pBEN vector served as controls. To assess whether *Rv1899c* conferred a survival advantage in host cells, THP-1-derived macrophage was infected with recombinant *M. smegmatis*-carrying *Rv1899c* or the vector alone. Infected macrophages were lysed at defined intervals, and bacterial survival was quantified by plating lysates on Middlebrook 7H10 agar. During the early phase of infection, recombinant *Rv1899c*-expressing *M. smegmatis* proliferated significantly, reaching a maximum survival of 156% at 12 h, whereas the control strain showed only marginal growth of 42% (Figure 3). Beyond 12 h, both recombinant and control strains exhibited a decline in viability, with bacterial counts approaching zero by 72 h, indicating complete clearance by the host macrophages.

### 2.3. Rv1899c Enhances the Resistance of M. smegmatis to Various Adverse Environments

To mimic the adverse conditions encountered within host macrophages, we tested the growth dynamics of recombinant *M. smegmatis* strains expressing *Rv1899c* under different stress environments. The primary mechanism by which *M. tuberculosis* persists within the host is its ability to survive the harsh conditions of the macrophage phagosome. This intracellular environment is defined by potent microbicidal stresses, specifically acidic pH and high levels of reactive oxygen species (ROS).

#### 2.3.1. Response to Acidic and Oxidative Stress

**Figure 4:**
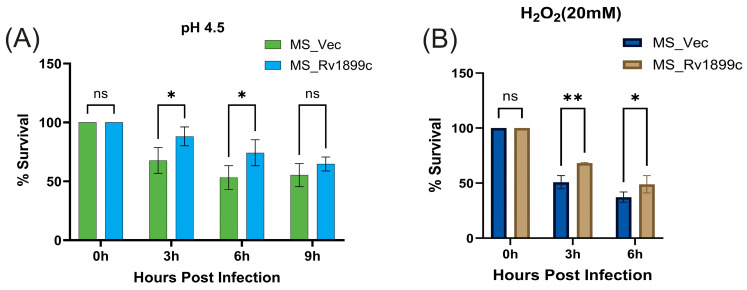
*Rv1899c* enhances *M. smegmatis* survival under stress conditions. Recombinant *M. smegmatis*-expressing *Rv1899c* (MS_*Rv1899c*) or vector control (MS_Vec) were exposed to stress conditions mimicking host environment, and bacterial survival was determined by CFU assay. (**A**) Acid stress assay: Bacteria were cultured in 7H9 medium adjusted to pH 4.5, and survival was monitored at the indicated time points. (**B**) Oxidative stress assay: Bacteria were treated with 20 mM H_2_O_2_, and survival was assessed at 0, 3, and 6 h post-exposure. Results are expressed as percentage survival relative to 0 h. Data represent mean ± SD from three independent experiments. Statistical significance was determined using one-way ANOVA with Šidák’s post hoc testing; *p* ≤ 0.05 (*), *p* ≤ 0.01 (**), ns: not significant.

To simulate phagosomal acidity, recombinant *M. smegmatis* strains were grown in MB 7H9 medium at pH 4.5. Following 3, 6, and 9 h of incubation, the MS_*Rv1899c* strain exhibited survival rates of 88.78%, 74.25%, and 64.70%, respectively, in contrast to the control MS_Vec strain, which displayed only 67.07%, 53.24%, and 55.28% survival (Figure 4A). The observation demonstrates that the protein Rv1899c confers a clear survival advantage in acidic conditions.

Similarly, to mimic oxidative stress, recombinant strains were exposed to 20 mM hydrogen peroxide for 3 and 6 h. The MS_*Rv1899c* strain exhibited survival rates of 68.2% and 49%, respectively, which were significantly higher than the 50.9% at 3 h and 37% at 6 h observed for the MS_Vec control (Figure 4B). Together with the acidic stress data, these findings indicate that *Rv1899c* enhances *M. smegmatis* resistance to both acidic and oxidative conditions without altering its baseline proliferative capacity.

#### 2.3.2. ROS Measurement in Macrophages Infected with *M. smegmatis* Vector Control and *Rv1899c*-Overexpressing *M. smegmatis*

**Figure 5:**
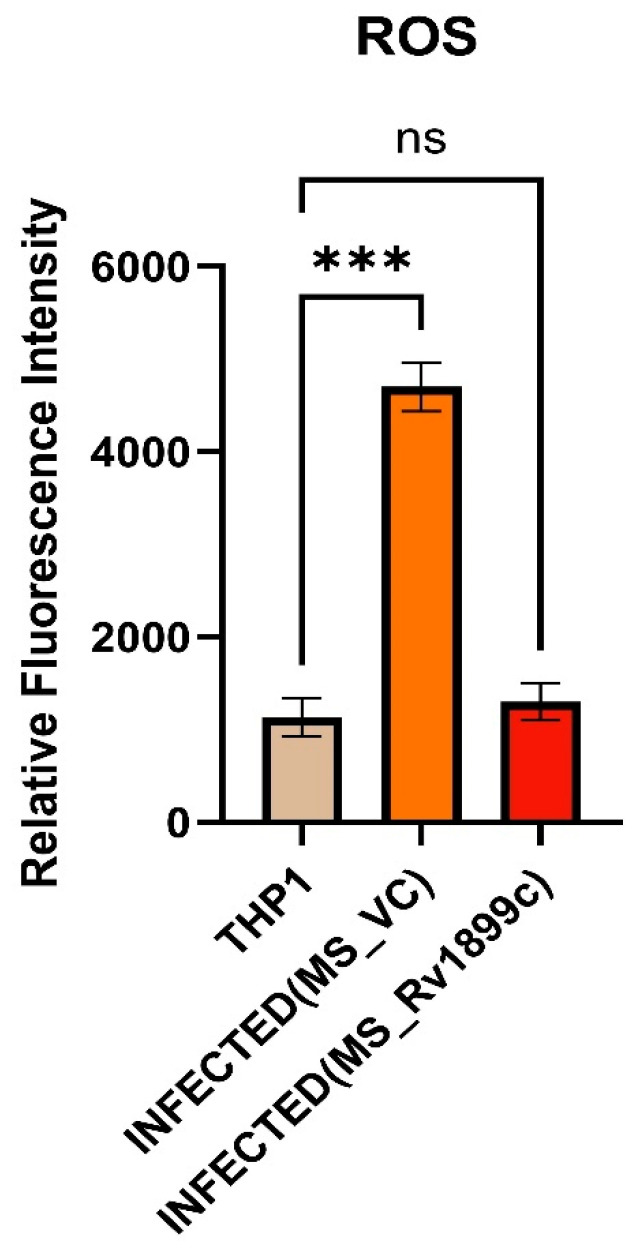
*Rv1899c* reduces ROS production in infected macrophages. THP-1-derived macrophages were either left uninfected (THP1) or infected with *M. smegmatis* vector control (MS_VC) or recombinant *M. smegmatis*-expressing *Rv1899c* (MS_*Rv1899c*). Intracellular ROS levels were measured at 24 h post-infection using a fluorescence-based ROS detection assay. Data are expressed as relative fluorescence intensity (RFI). Results represent mean ± SD from three independent experiments. Statistical significance was determined using one-way ANOVA with Šidák’s post hoc testing; *p* ≤ 0.001 (***), ns: not significant.

The role of *Rv1899c* in modulating oxidative stress during infection was examined by measuring reactive oxygen species (ROS) levels in THP-1-derived macrophages. ROS production was quantified using the fluorescent probe DCFDA. THP-1 cells were infected either with *M. smegmatis* carrying the empty pBEN vector (MS_Vec) or with *M. smegmatis*-overexpressing *Rv1899c* (MS_*Rv1899c*). Following infection, cells were incubated with DCFDA, and fluorescence intensity was recorded using a fluorescence spectrophotometer as a measure of intracellular ROS levels. Analysis revealed a significant reduction in ROS production in macrophages infected with MS_*Rv1899c* compared with those infected with the control strain (Figure 5). This suggests a potential role for this protein in modulating the host oxidative stress response during mycobacterial infection.

### 2.4. Rv1899c Suppresses the Secretion of Pro-Inflammatory Cytokines, Autophagy Gene ATG5, Baclin, LC3 and Reduces the Populations of M1 Macrophages upon Infection

**Figure 6:**
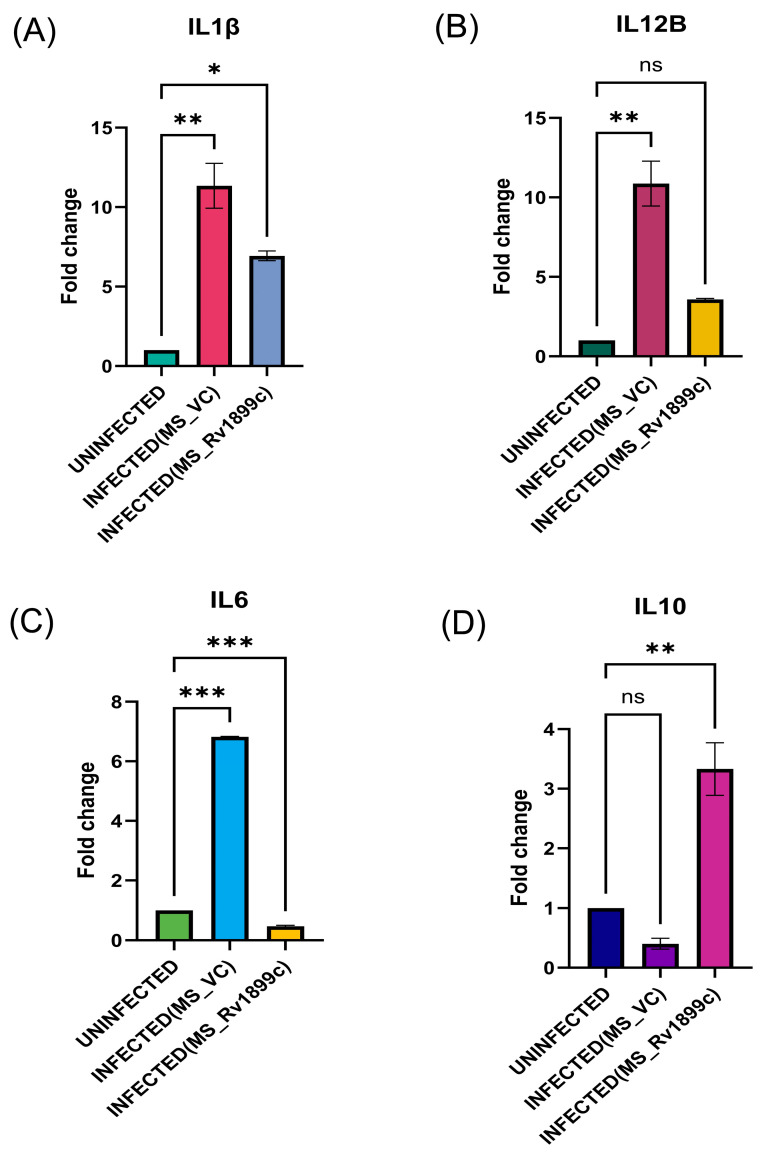
THP-1-derived macrophages were left uninfected or infected for 24 h with wild-type M. smegmatis carrying the empty vector (MS_VC) or recombinant M. smegmatis overexpressing *Rv1899c* (MS_*Rv1899c*). Cytokine mRNA levels were quantified by qPCR and normalized to β-actin expression. (**A**) IL1β and (**B**) IL12B were elevated in MS_VC-infected macrophages but reduced upon MS_*Rv1899c* infection. (**C**) IL6 expression was strongly induced by MS_VC and suppressed by MS_*Rv1899c*, while (**D**) IL10 was significantly upregulated in MS_*Rv1899c*-infected cells. Data represent mean ± SD from three independent experiments performed in triplicate. Statistical significance was determined using one-way ANOVA with Šidák’s post hoc test (*p* ≤ 0.05 (*), *p* ≤ 0.01 (**), *p* ≤ 0.001 (***), ns: not significant).

**Figure 7:**
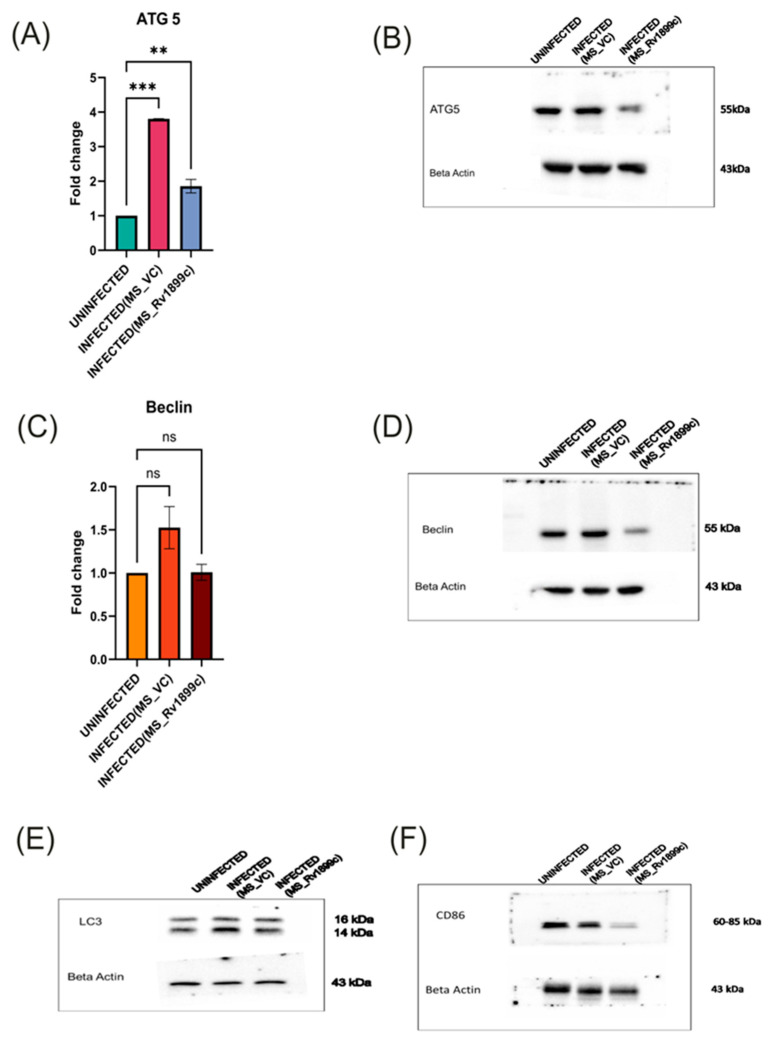
Modulation of autophagy and macrophage polarisation markers in THP-1 macrophages infected with recombinant *M. smegmatis*-overexpressing *Rv1899c*. THP-1-derived macrophages were left uninfected or infected for 24 h with wild-type *M. smegmatis* carrying an empty vector (MS_VC) or with recombinant *M. smegmatis*-expressing *Rv1899c* (MS_*Rv1899c*). Gene expression was analyzed by qPCR (normalized to β-actin), and protein levels were assessed by Western blotting. Data represent the mean ± SD of three independent experiments. Statistical significance was determined using one-way ANOVA with Šidák’s post hoc testing (** *p* < 0.01; *** *p* < 0.001; ns, not significant). (**A**,**B**) ATG5: qPCR showed a significant increase in *ATG5* mRNA expression upon MS_VC infection compared with uninfected cells, whereas MS_*Rv1899c* infection resulted in reduced expression. Western blot analysis confirmed corresponding changes in ATG5 protein (~55 kDa). (**C**,**D**) Beclin: qPCR analysis revealed a downregulation of Beclin 1 mRNA levels, which was further supported by Western blot data demonstrating a significant reduction in Beclin 1 protein (~55 kDa) abundance in the infected group relative to the uninfected control. (**E**) LC3: Western blotting showed changes in LC3-I (16 kDa) and LC3-II (14 kDa) levels in infected macrophages. (**F**) CD86: Western blot analysis revealed modulation of CD86 expression (60–85 kDa) following infection. β-actin (~43 kDa) was used as the loading control.

To assess the impact of *Rv1899c* on host immune responses, cytokine gene expression was measured in infected THP-1-derived macrophages by qPCR. Macrophages infected with the recombinant strain overexpressing *Rv1899c* (MS_*Rv1899c*) produce significantly lower levels of the pro-inflammatory cytokines IL12B, IL-1β, IL-6 and higher levels of anti-inflammatory cytokine IL-10 (Figure 6A–D) compared with those infected with the vector control strain (MS_Vec) after 24 h of infection. ATG5, a key regulator of autophagosome formation and autophagic flux, along with Beclin-1, which initiates autophagosome nucleation, showed reduced expression in macrophages infected with MS_*Rv1899c*, as demonstrated by qPCR and Western blot analyses (Figure 7A–D). Importantly, the conversion of LC3-I to LC3-II was also significantly reduced in MS_*Rv1899c*-infected macrophages, confirming a suppression of autophagosome formation (Figure 7E) Collectively, these results indicate that *Rv1899c* suppresses pro-inflammatory cytokine responses and autophagy, upregulates anti-inflammatory cytokines, thereby creating a cellular environment that favours mycobacterial persistence.

Further analysis revealed that the immunosuppressive activity of *Rv1899c* also extends to macrophage polarization. While macrophage populations typically comprise both pro-inflammatory M1 and anti-inflammatory M2 subsets, infection with MS_*Rv1899c* resulted in a marked reduction in the M1 population. This shift was supported by Western blot analyses, which showed decreased expression of canonical M1 marker CD86 (Figure 7F). The suppression of M1 polarization highlights a sophisticated immune evasion strategy whereby *Rv1899c* dampens pro-inflammatory responses and promotes an intracellular environment favourable for mycobacterial survival.

### 2.5. Binding Affinity and Interaction Analysis of Rv1899c with 3-Aminobenzamide

**Figure 8:**
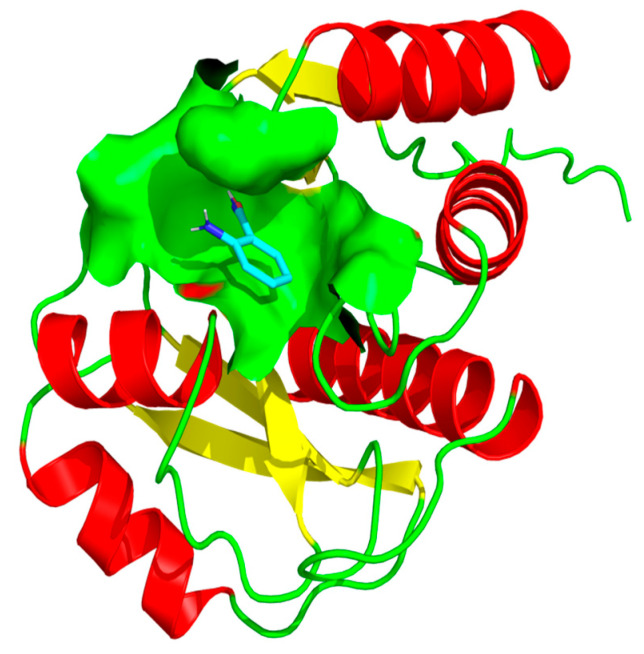
Surface representation of the 3-aminobenzamide binding pocket within the Rv1899c structure. The figure illustrates the interaction of 3-aminobenzamide (shown as stick representation) within the active site cavity of the protein. The molecular surface (light green) highlights the binding pocket that accommodates 3-aminobenzamide, depicting the spatial orientation and accessibility of the ligand within the active site region.

**Figure 9:**
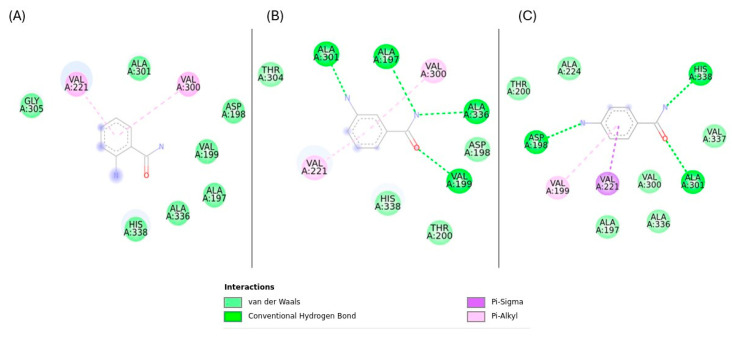
Key molecular interactions of Rv1899c with aminobenzamide isomers (**A**) 2-aminobenzamide, (**B**) 3-aminobenzamide, (**C**) 4-aminobenzamide.

Rv1899c exhibited sequence similarity to the human ADP-ribosyl polymerase PARP14, which contains macrodomains in addition to the catalytic ADP-ribosyl transferase domain. Based on this similarity, we employed 3-aminobenzamide (3-AB), a well-characterized inhibitor of ADP-ribosyl polymerases, to assess its potential effect on Rv1899c activity [13]. Molecular docking simulations were performed to elucidate the interaction patterns of three aminobenzamide isomers with the Rv1899c macrodomain. From the results, it is identified that 3-aminobenzamide exhibited the strongest interaction with an affinity score of −5.754 kcal/mol. (Figure 8) shows the surface representation of binding pocket amino acids in 3-aminobenzamide complex. Subsequent molecular interaction analysis revealed the key amino acids involved in stabilizing the drug target complexes as presented in (Figure 9).

3-aminobenzamide complex was stabilized by the formation of hydrogen bonds with VAL197, VAL199, ALA301, and ALA336 residues, Pi–alkyl interactions with VAL221, and VAL300 residues. 4-aminobenzamide showed a slightly lower binding affinity of −5.421 kcal/mol. This complex formed three hydrogen bonds with ASP198, ALA301, and HIS338 residues, Pi–Alkyl interaction with VAL199, and Pi–Sigma interactions with VAL221 residues. 2-aminobenzamide complex was stabilized primarily with Pi–Alkyl interactions with VAL221 and VAL300 residues. This complex showed the lowest binding energy of −5.12 kcal/mol. These results suggest that 3-aminobenzamide is the most promising ligand with favourable interactions, likely due to a stable interaction facilitated by a greater number of hydrogen bonds.

### 2.6. Molecular Dynamics Simulation Analysis

A 200 ns molecular dynamics (MD) simulation was performed to assess the binding stability of 3-aminobenzamide–Rv1899c complex. This method gives insights beyond the molecular docking studies by quantifying the dynamic changes in protein–ligand complex in a solvated system for a specified duration. The trajectories obtained from the simulation were further analyzed.

**Figure 10:**
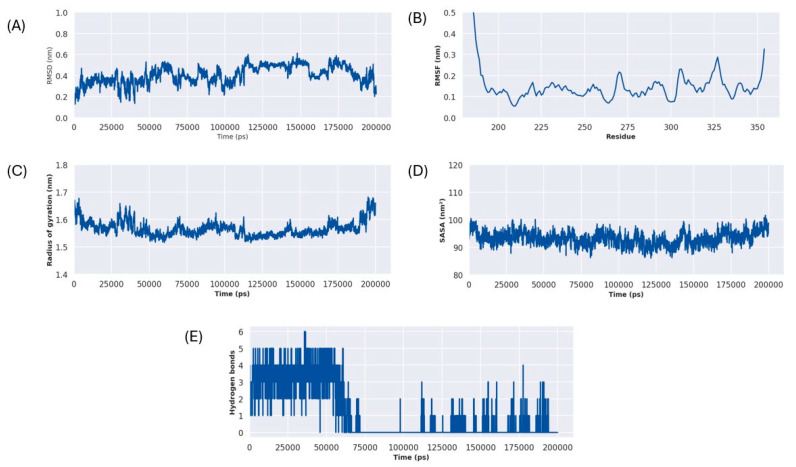
3-aminobenzamide–Rv1899c complex MD simulation metrics (**A**) RMSD, (**B**) RMSF, (**C**) RG, (**D**) SASA, (**E**) H-Bonds.

The overall stability and structural integrity of protein backbone atoms during the simulation run was quantified by using Root-Mean-Square Deviation (RMSD), as depicted in (Figure 10A). The system showed an initial fluctuation for a period of 50ns, after which RMSD fluctuations became stable around an average value of approximately 0.4nm. This indicates the stable conformation of protein molecule without significant structural deviations. Root Mean Square Fluctuations (RMSF) was calculated to understand the dynamic behaviour of protein molecules at the residue level and to identify the flexible regions in the protein structure. Most residues in the structure showed low RMSF values below 0.2 nm, while a few loop regions showed slightly higher flexibility, as shown in (Figure 10B). The most significant fluctuations were observed in the terminal regions. In addition, the residues present in the loop regions such as residues 324–328 showed RMSF values between 0.2 and 0.3.

Solvent Accessible Surface Area (SASA), shown in (Figure 10C), and the Radius of Gyration (Rg), shown in (Figure 10D), were calculated to assess the compactness of the protein–ligand complex. The Rg plot depicts consistent values throughout the simulations averaging around 1.58 nm. This observation is further supported by the SASA plot, which remained stable around an average SASA value of 95 nm^2^. These results suggest that protein–ligand complex remained compact and did not undergo any major unfolding events during the simulation run.

The stability of the protein–ligand interaction was monitored by assessing the frequency of hydrogen bond formed between ligands and protein throughout the simulation run, as shown in (Figure 10E). For the first 65ns, the complex showed a significant number of hydrogen bonds between 1 and 6. After this period, from 65 ns to 110 ns, the initially observed key interactions were completely lost. However, after this period from 110ns the hydrogen bonds were reformed, fluctuating between 0 and 3 bonds, indicating a less tightly bound conformation, which still maintains a consistent contact with the protein.

### 2.7. Binding Free Energy Calculation Using MM/GBSA

**Figure 11:**
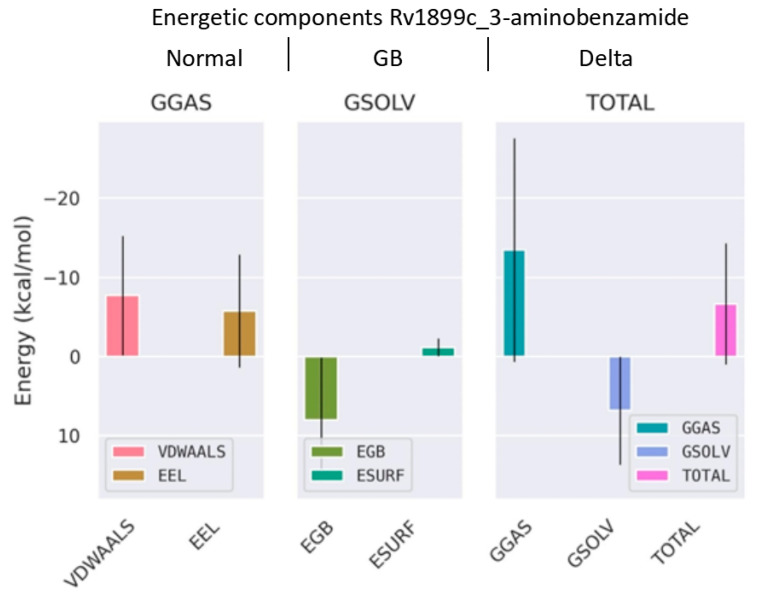
Binding free energy decomposition from MM/GBSA analysis using gmx_MMPBSA tool version 1.6.4. The plot shows the contributions of van der Waals (VDWALLS), electrostatic (EEL), polar solvation (EGB), and nonpolar solvation (ESURF) energies to the overall binding free energy (TOTAL) of the complex.

The binding free energy (ΔGbind) of the 3-aminobenzamide–Rv1899c complex was calculated using MM/GBSA method. This analysis revealed a favourable binding free energy of −6.66 kcal/mol shown in (Figure 11). This analysis revealed that this binding is primarily driven by strong, favourable van der Waals interaction energy (ΔEvdw) of −7.71 kcal/mol, and electrostatic interaction energy (ΔEele) of −5.76 kcal/mol. The solvation energy terms such as polar solvation energy (ΔEgb) and nonpolar energy (ΔEsurf) depict the energetics of desolvating the binding surfaces. ΔEgb is positive for the complex with a value of 7.97 kcal/mol and ΔEsurf showed a value of −1.16 kcal/mol. The positive nonpolar solvation energy indicates the desolvation of system upon protein–ligand bonding. Overall, this decomposition analysis of energetic terms reveals that 3-aminobenzamide forms an energetically favourable complex with the Rv1899c macrodomain.

### 2.8. Inhibitor Treatment for Rv1899c and Survival Assays

**Figure 12:**
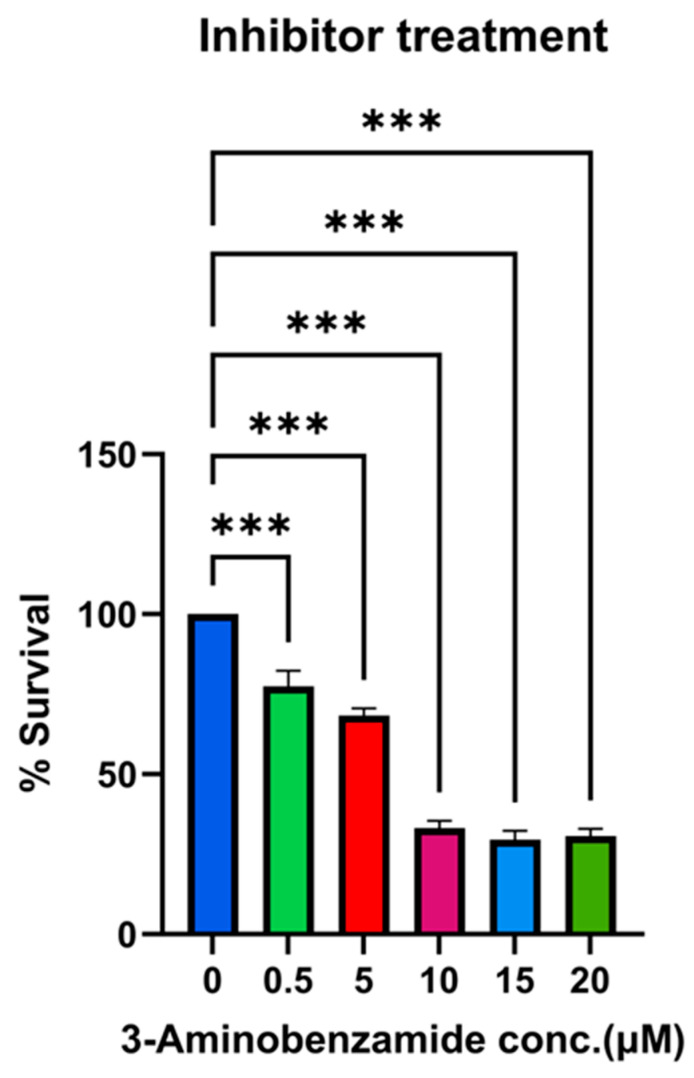
Dose-dependent reduction in bacterial survival by 3-aminobenzamide (3-AB) treatment. THP-1-derived macrophages were infected with recombinant *M. smegmatis* overexpressing Rv1899c for 24 h and treated with increasing concentrations of the PARP inhibitor 3-aminobenzamide (3-AB) (0–20 µM). Intracellular bacterial survival was determined by lysing macrophages, plating on 7H10 agar, and enumerating CFUs after incubation. Survival at each concentration is expressed as a percentage relative to the untreated control (0 µM). Data represent the mean ± SD of three independent experiments performed in triplicate. Statistical significance was determined using one-way ANOVA followed by Šidák’s post hoc test (*** *p* < 0.001).

**Figure 13:**
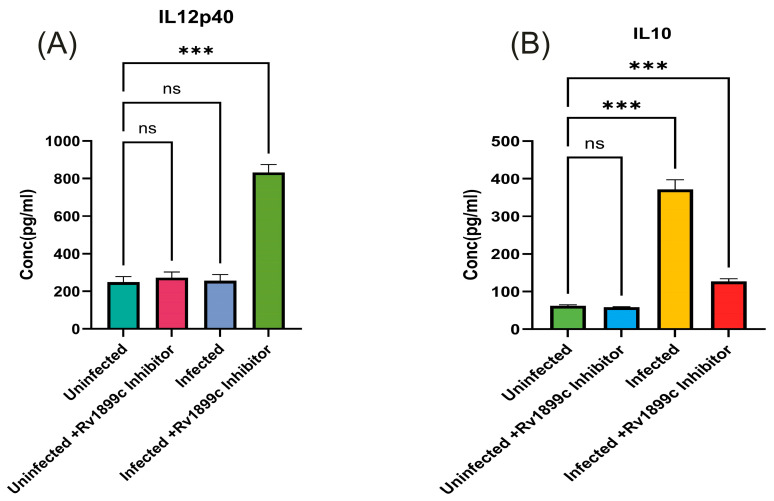
Rv1899c-Mediated Modulation of Pro- and Anti-inflammatory Cytokines in Infected Macrophages and the Restorative Effect of Its Inhibition. THP-1-derived macrophages were either left uninfected or infected with recombinant *M. smegmatis*-overexpressing *Rv1899c*, followed by treatment with Rv1899c inhibitor (3-aminobenzamide) for 24 h. Supernatants were collected and cytokine concentrations were measured by ELISA. (**A**) The levels of IL-12p40 were quantified. Macrophages infected with Rv1899c-overexpressing strains showed significantly reduced IL-12p40 production compared to uninfected controls, whereas treatment of infected macrophages with the Rv1899c inhibitor (3-AB) resulted in a significant elevation of IL-12p40 levels compared to untreated infected controls.. (**B**) Levels of IL-10 were quantified. Macrophages infected with Rv1899c-overexpressing strains showed significantly elevated IL-10 production compared to uninfected controls. Infected macrophages exhibited markedly increased IL-10 secretion, which was significantly reduced upon treatment with the Rv1899c inhibitor (3-AB). Data are presented as mean ± SD from three independent experiments. Statistical significance was determined by one-way ANOVA with Šidák’s post hoc testing; *** *p* ≤ 0.001, ns = not significant.

To evaluate the contribution of Rv1899c enzymatic activity to intracellular bacterial persistence, we used 3-aminobenzamide (3-AB), a broad-spectrum inhibitor of ADP-ribosylating enzymes. THP-1-derived macrophages infected with MS_*Rv1899c* were treated with increasing concentrations of 3-AB (0.5–20 μM), and bacterial viability was assessed by plating serial dilutions of macrophage lysates on 7H10 agar. Inhibitor treatment resulted in a dose-dependent reduction in bacterial survival, with significant decreases observed at 10 μM and above (~33.2 ± 2.2% at 10 μM, 29.5–30.6% at 15–20 μM, 24 hpi) (Figure 12). Based on these findings, 10 μM 3-AB was selected for subsequent experiments. The levels of IL-12p40 and IL-10 were quantified to assess the impact of Rv1899c on cytokine modulation during infection. Macrophages infected with Rv1899c-overexpressing strains exhibited a significant reduction in IL-12p40 production compared to uninfected controls, indicating suppression of pro-inflammatory signaling. In contrast, treatment of infected macrophages with the Rv1899c inhibitor led to a marked increase in IL-12p40 levels relative to untreated infected controls. Conversely, IL-10 production was significantly elevated in macrophages infected with Rv1899c-overexpressing strains compared to uninfected controls, suggesting a shift toward an anti-inflammatory response. Notably, treatment with the Rv1899c inhibitor significantly reduced IL-10 secretion, restoring a more balanced cytokine profile (Figure 13A,B). These effects may represent a synergistic outcome of Rv1899c’s ADP-ribosylating activity and the concomitant inhibition of host PARPs by 3-AB, as both are known to modulate PARP-dependent transcriptional and inflammatory pathways. This dual interference could collectively attenuate host PARP signalling, leading to the observed reversal of cytokine responses and impaired bacterial persistence.

### 2.9. Combinatorial Treatment for Rv1899c Inhibitor and HDAC1 Inhibitor

**Figure 14:**
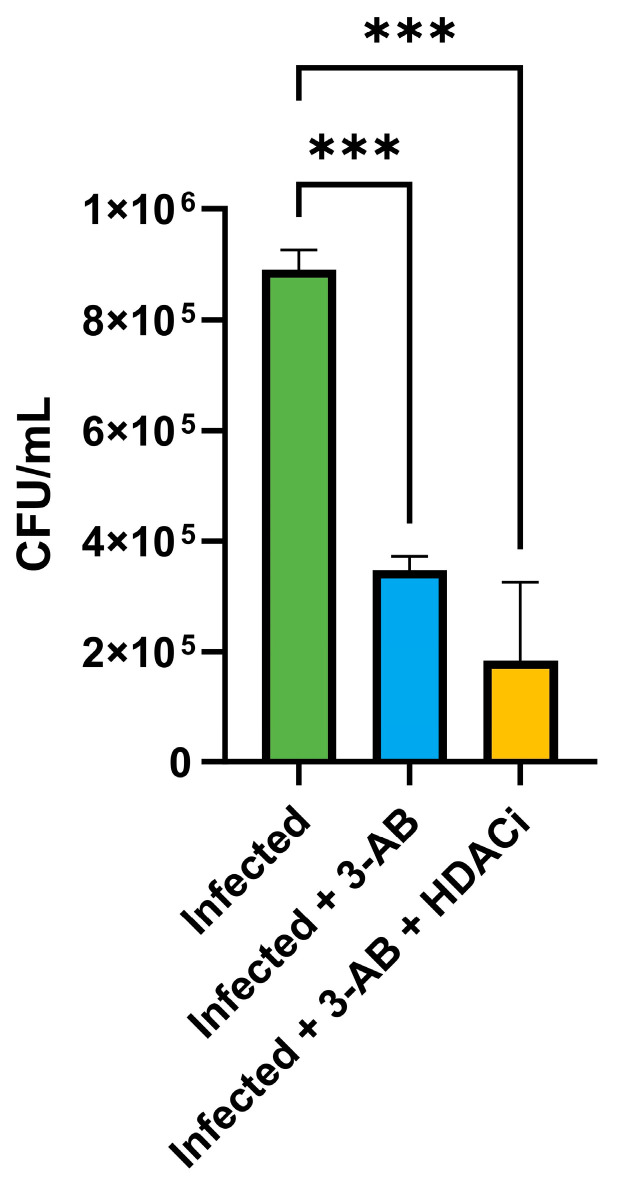
Effect of Rv1899c and HDAC1 inhibitors on bacterial survival and cytokine responses in macrophages. THP-1-derived macrophages were infected with recombinant *M. smegmatis*-overexpressing *Rv1899c* and treated with Rv1899c inhibitor 3-aminobenzamide (3-AB) alone or in combination with HDAC1 inhibitor (HDACi) CI994 for 24 h. Intracellular bacterial viability was determined by CFU enumeration. Treatment with 3-AB significantly reduced bacterial survival, which was further decreased by combinatorial treatment with 3-AB and HDACi. Data are presented as mean ± SD from three independent experiments. Statistical significance was determined by one-way ANOVA with Šidák’s post hoc testing; *** *p* ≤ 0.001.

The ability of 3-AB to reduce intracellular bacterial survival suggested that it could potentiate the activity of other therapeutic agents. In our previous work, we demonstrated the efficacy of the HDAC inhibitor CI994 in controlling *Mycobacterium tuberculosis* infection in macrophages [12]. To test for synergy, THP-1-derived macrophages were infected with MS_*Rv1899c* and subsequently treated with 3-AB in combination with CI994. After 24 h, CFU enumeration revealed a marked reduction in intracellular bacteria, with the combination treatment achieving ~79% killing, compared with ~61% for 3-AB alone (Figure 14).

## 3. Discussion

Collectively, our findings identify *Rv1899c* as a potent immunomodulatory effector that enables mycobacteria to persist within macrophages by dampening multiple arms of the host defence. Macrophage infected with *Rv1899c*-expressing *M. smegmatis* secreted reduced levels of pro-inflammatory cytokines (IL12B, IL-1β, IL-6) and exhibited transcriptional repression of IL-12B, thereby weakening Th1 priming and IFN-γ-mediated macrophage activation [12].

The ability of *M. tuberculosis* to establish a persistent infection largely depends on how effectively it withstands the host’s early immune defences, especially the acidic environment and oxidative stress (ROS) within macrophage phagosomes. Our results show that expression of *Rv1899c* markedly improves the survival of *M. smegmatis* under both acidic and oxidative stress, highlighting its pivotal role in intrinsic stress resistance. Notably, *Rv1899c*-expressing bacteria also significantly lowers intracellular ROS levels in infected host cells. This enhanced resilience is not just an in vitro observation, it represents the critical first step in pathogenesis, allowing the bacteria to survive, adapt, and multiply before initiating more complex mechanisms of immune modulation, including altering macrophage polarization (M1/M2 balance) and suppressing autophagy.

In addition, these cells displayed downregulation of the autophagy gene ATG5, Beclin 1 and reduced LC3-II accumulation and diminished expression of M1 polarization markers, consistent with a shift toward an anti-inflammatory phenotype. Together, these alterations create a permissive intracellular niche that favours bacterial survival. Mechanistically, our data support the notion that Rv1899c functions as an ADP-ribosylating effector that co-opts host epigenetic machinery, including the HDAC1-ZBTB25-Sin3a complex, to repress inflammatory gene expression and autophagy pathways.

Positioning *Rv1899c* within the broader repertoire of *M. tuberculosis* virulence factors underscores that immune modulation by individual bacterial effectors is both common and multifactorial. For example, ESAT-6 (EsxA, Rv3875) is a prototypical secreted virulence protein that disrupts host membranes, modulates inflammasome activity, and interferes with autophagic flux in macrophages; ESAT-6-mediated membrane perturbation and signalling rewiring ultimately impair xenophagic clearance of bacilli [10,14,15]. Likewise, the Eis protein (Rv2416c) acetylates host substrates to inhibit JNK-dependent autophagy, reduce ROS generation and suppress pro-inflammatory signalling, thereby enhancing intracellular persistence [8]. These examples highlight that suppression of cytokines production, blockade of autophagic pathways, and attenuation of oxidative killing represent recurrent strategies employed by diverse *M. tuberculosis* effectors—paralleling the multiple activities we have identified for *Rv1899c* [16,17].

Multiple *M. tuberculosis* effectors directly interfere with autophagy at distinct stages, underscoring that this pathway is a major bacterial target. The serine/threonine kinase PknG (Rv0410c) disrupts autophagosomes maturation by engaging host small GTPases such as RAB14, thereby blocking endo-lysosomal trafficking and causing an accumulation of immature autophagosomes that shield bacilli from lysosomal degradation [18]. Similarly, the secreted phosphatase SapM (Rv3310) dephosphorylates PI3P, impairing phagosomal maturation and preventing autophagosome-lysosome fusion [19]. Members of the PE_PGRS family, including PE_PGRS47 and PE_PGRS20, inhibit autophagy initiation by interacting with Rab1A and concurrently block MHC class II antigen presentation through disrupted autophagic trafficking of mycobacterial antigens [19,20]. Together, these examples illustrate the range of strategies kinase and phosphatase-mediated modification, acetyltransferase activity, interference with host GTPases, and recruitment of host repressor complexes by which *M. tuberculosis* converges on autophagy suppression. The downregulation of ATG5, Beclin and the reduction in LC3-dependent autophagy observed with Rv1899c align with this broader pattern, reinforcing the notion that inhibition of host degradative pathways is central to mycobacterial persistence.

Interference with cytokines signalling and antigen presentation is another recurring strategy of *M. tuberculosis*. The tyrosine phosphatase PtpA (Rv2234) prevents phagosome acidification by excluding the host V-ATPase and dephosphorylating key host substrates, thereby blocking phagosome maturation with downstream effects on antigen processing and innate immune signalling [9]. The surface-exposed chaperonin Cpn60.2 (GroEL2, Rv0440), along with related chaperonins, modulates cytokine production and dampens inflammatory recognition, reducing macrophage activation [21]. Collectively, these effectors converge on suppression of TNF-α, IL-1β, IL-6, and IL-12, while simultaneously perturbing antigen presentation. The observation that *Rv1899c* similarly reduces these cytokines, including *IL-12B*, situates it firmly within the canonical immune subversion repertoire of *M. tuberculosis*.

Mechanistically, Rv1899c’s interaction with HDAC1 and ZBTB25 is partially noteworthy, as epigenetic silencing of inflammatory promoters offers a durable mode of immune suppression that does not depend on continuous effector secretion. Recruitment of HDAC1 is a recognized bacterial tactic: histone hypoacetylation at pro-inflammatory loci such as IL-12 leads to transcriptional silencing, weakened Th1 priming, and reduced antimicrobial activation [12]. This nuclear strategy contrasts with effectors like Eis or SapM, which act in the cytosol or phagosomal membrane to disrupt kinase cascades or lipid signalling-distinct mechanism that nonetheless converge on dampening host defences. The predicted ADP-ribosyltransferase activity of Rv1899c may provide an enzymatic means of stabilizing the HDAC1-ZBTB25 complex at target promoters or modulating upstream signalling pathways that drive cytokine induction. In this sense, *Rv1899c* exemplifies how *M. tuberculosis* effectors harness diverse post-translational modifications—acetylation, phosphorylation, dephosphorylation, and ADP-ribosylation—to reprogram host immunity.

In addition to the cellular and molecular evidence for the immunomodulatory role of *Rv1899c*, our structural analyses further reinforce its potential as a host-targeted effector. The three-dimensional structure of the Rv1899c macrodomain predicted by AlphaFold was subjected to rigorous validation, including MolProbity and PROCHECK assessments, which confirmed stereochemical integrity and high-quality geometry with no disallowed residues [22]. These findings provide confidence that the predicted fold is reliable for downstream functional inferences. Molecular docking simulations with aminobenzamide isomers revealed that 3-aminobenzamide exhibited the strongest binding affinity, stabilized through multiple hydrogen bonds and hydrophobic interactions, suggesting that the Rv1899c macrodomain harbours a druggable binding pocket [23]. Molecular dynamics simulations further supported the stability of this complex, with consistent RMSD, Rg, and SASA values, as well as dynamic but persistent hydrogen-bonding interactions, indicative of a conformationally stable ligand −6.66kcal/mol [24]. Together, these structural and energetic insights not only validate the Rv1899c macrodomain as a functionally stable fold but also suggest that it could act as an ADP-ribosylating effector capable of engaging host nuclear machinery. These findings bridge the biochemical evidence of immune suppression with structural predictions, positioning Rv1899c as a promising candidate for therapeutic targeting [25,26].

Blocking the activity of Rv1899c emerges as a compelling strategy to control mycobacterial infection. Our findings show that treatment with 3-aminobenzamide (3-AB) markedly reduced the intracellular survival of *M. tuberculosis* and helped restore the host’s natural antimicrobial defences. Computational analysis including molecular docking and molecular dynamics simulations strongly support that 3-AB binds directly and specifically to the Rv1899c macrodomain. The observed decline in bacterial load and reversal of immune suppression point to the inhibition of the enzyme’s ADP-ribosylating activity as the main mechanism behind its antibacterial effect. 3-AB is also a well-known broad-spectrum inhibitor of host Poly (ADP-ribose) polymerases (PARPs), such as PARP1. Therefore, its therapeutic impact likely arises from a combination of actions blocking a crucial bacterial virulence factor while simultaneously influencing host PARP-mediated pathways. This dual mode of activity offers an intriguing therapeutic opportunity.

## 4. Materials and Methods

### 4.1. Processing and Validation of Protein Structure

The three-dimensional structure of the uncharacterized protein Rv1899c (UniProt ID: P9WK29) was obtained from the AlphaFold Protein Structure Database [27,28], shown in Figure 1A. From this complete structure containing 359 residues, the macro or A1pp domain, corresponding to residues 179–354, was isolated and extracted; the resulting macrodomain structure is shown in Figure 1B. This procedure was performed using PyMol molecular graphics system, version 2.5.0 [29]. Prior to subsequent analysis, the resulting structure was validated using MolProbity (https://molprobity.biochem.duke.edu/, Accessed on 5 September 2025) [30] and PROCHECK (https://saves.mbi.ucla.edu/, Accessed on 6 September 2025) [23] web server to ensure the structural integrity and stereochemical quality.

### 4.2. Molecular Docking

Molecular docking studies were executed on the Rv1899c macrodomain 3D structure to predict the binding conformations and binding energy of three ligands (2-aminobenzamide, 3-aminobenzamide, 4-aminobenzamide). The SDF files containing the atomic coordinates of ligands were obtained from PubChem database [31]. These ligand coordinate files were prepared using OpenBabel (https://www.cheminfo.org/Chemistry/Cheminformatics/FormatConverter/index.html, Accessed on 7 September 2025) [32] and AutoDock [33] Tools (ADT), version 1.5.7p1. This preparation involves defining rotatable bonds to ensure the conformational flexibility of the ligand molecules during the docking process. The receptor structure was also prepared using ADT; this includes adding nonpolar hydrogens, adding Kollman partial charges to the protein atoms, and finally saving the structure in PDBQT format.

AutoDock Vina molecular docking software, version 1.2.3 [34] was utilized for performing the molecular docking simulations. Blind docking method was utilized for defining the search space, a grid box with size of 84 Å × 112 Å × 88 Å was centred on the protein structure in a manner that it covers the entire protein molecule. Exhaustiveness parameter was set to be 25 to ensure a comprehensive exploration of possible ligand binding conformations. For each ligand, the conformation exhibiting lowest binding energy value (kcal/mol) was selected as the most favourable binding conformation. These conformations were then visualized and analyzed using PyMOL and Discovery Studio Visualizer, version 25.1.0.24284 to identify key molecular interactions such as hydrogen bonds and van der Waals interaction.

### 4.3. Molecular Dynamics Simulations

Molecular dynamics simulations were performed to evaluate the dynamic stability of 3-aminobenzamide–Rv1899c macrodomain complex selected based on molecular docking studies. All simulations utilized GROMACS 2025.2 software [35,36]. The CHARMM36 all-atom force field [37,38] was utilized for approximating the calculations necessary for the protein–ligand complex simulations. The CGenFF servrer (https://app.cgenff.com/homepage, Accessed on 10 September 2025) was utilized for generating the ligand topology and parameter files [39,40,41]. Each protein–ligand complex was then aligned in the centre region of a dodecahedron box, maintaining a minimum distance of 1.0 nm to the box boundaries. Next, the simulation system was solvated with TIP3P water molecules. gmx genion tool, version 2025.2 was utilized for neutralizing the system with Na^+^ and Cl^−^ ions. This process is carried out to achieve a physiological salt concentration of 0.15 M. This resulted in addition of 4 Na^+^ ions to the system.

Following solvation, the complex underwent energy minimization using steepest descent algorithm for 5000 steps. The system was further subjected to NVT equilibrium at 300 K using V-rescale thermostat [42]. NPT equilibrium was performed on the complex to stabilize the pressure at 1 bar using Berendsen barostat [43]. Both equilibration phases were conducted out for a period of 100 ns with positional restraints on heavy atoms. Subsequently, a 200 ns MD simulation production proceeded freely without restraints at 300K temperature and 1 bar pressure under the NPT ensemble. LINCS algorithm [44] was utilized for constraining hydrogen bonds. Particle mesh Ewald (PME) [45] was applied for computing the long-range electrostatics. Leap-frog integration algorithm [46] facilitated the integration of trajectories with a time-step of 2fs. The molecular dynamic trajectories were examined using GROMACS. Analysis included parameters such as gmx rms, gmx rmsf, gmx gyrate, gmx sasa, and gmx hbond. Matplotlib, version 3.10.0 [47] and seaborn, version 0.13.2 [48] python libraries were utilized for plotting the resulting metrics.

### 4.4. MM/GBSA Analysis

Molecular Mechanics/Generalized Born Surface Area (MM/GBSA) method [25] was employed to calculate the binding free energy of the 3-aminobezamide–Rv1899c complex. The gmx_MMPBSA tool, version 1.6.4 [49,50] was employed to estimate the binding free energies, using 4000 frames uniformly sampled at 50 ps intervals from the 200 ns molecular dynamics production trajectory.

Binding free energy (ΔGbind) calculations for the complex were performed using the following equation:

ΔGbind ≈ ⟨Ecomplex ⟩ − (⟨Ereceptor ⟩ + ⟨Eligand⟩)(1)

in which each term can be broken down as

E = ΔEmm + ΔGsolv(2)

where ΔEmm is change in molecular mechanistic energy (sum of ΔEvdw, and ΔEele), ΔGsolv is the change in solvation free energy (sum of ΔEgb and ΔEsurf). The energy calculated over all the sampled frames was averaged to determine the ΔGbind of the complex. The final result was visualized using gmx_MMPBSA_ana tool, version 1.6.4.

### 4.5. Cloning and Expression of Rv1899c

The *Rv1899c* gene was PCR-amplified from *M. tuberculosis* H37Rv genomic DNA using primers designed to incorporate BamHI and HindIII restriction sites, along with an N-terminal FLAG tag for subsequent protein detection. The forward primer (pBENADF) was 5′-GATCGGATCCATGGACTACAAAGACGATGACGACAAGATGTCCCGGGCTGCCGGGTTGCCC-3′ and the reverse primer (pBENADR) was 5′-GATCAAGCTTCTACCGTCGAGCGGTATCTTCT-3′. The PCR was carried out with an initial denaturation at 95 °C, 3 min, succeeded by 30 cycles of 95 °C, 30 s, 60.7 °C, 30 s, and 72 °C, 1 min, and concluded with a final elongation step at 72 °C, 5 min. The purified PCR product and the constitutive mycobacterial expression vector pBEN were then digested with BamHI and HindIII and ligated to generate the recombinant construct pBEN-*Rv1899c*.

The pBEN vector is a shuttle plasmid designed for constitutive expression in *Mycobacterium* spp. It contains the hsp60 promoter from *Mycobacterium bovis* BCG, which drives high-level, constitutive gene expression in both *M. smegmatis* and *M. tuberculosis*. The vector carries a kanamycin resistance gene (Kan^R^) for selection in both *E. coli* (50 µg/mL) and *Mycobacterium*

The recombinant plasmid was delivered to *M. smegmatis* mc^2^155 via electroporation. Mid-log phase cultures were harvested, washed with sterile 10% glycerol, and mixed with approximately 1 µg of the ligated plasmid DNA. Electroporation was performed using Gene pulser system (Bio-Rad, Hercules, CA, USA) set at 2.5 kV, 200 Ω, and 25 µF. Following a 3–4 h recovery period in Middlebrook 7H9 culture medium with added 0.05% Tween-80 and glycerol at 37 °C, cells were plated on 7H9 agar containing kanamycin (25 µg/mL) for selection. Colonies appearing after 3–5 days were screened by colony PCR to confirm successful integration of *Rv1899c*.

### 4.6. Cell Culture and Differentiation of THP-1 Monocytes

THP-1 monocytes, human monocytic cell line (ATCC-TIB-202), were cultured in RPMI 1640 medium (Gibco, Thermo Fisher Scientific, Waltham, MA, USA) supplemented with 10% heat-inactivated Fetal Bovine Serum (FBS; Gibco) and 1% penicillin-streptomycin (Invitrogen, Waltham, MA, USA) solution. Cells were cultured at 37 °C in a humidified 5% CO_2_ incubator. Differentiation into macrophage-like cells was induced by exposing THP-1 monocytes to 20 ng/mL phorbol-12-myristate-13-acetate (PMA; Sigma-Aldrich, Saint Louis, MO, USA) for 24 h. Successful differentiation was confirmed by monitoring adherence and morphological changes using phase-contrast microscopy.

### 4.7. Infection of THP-1-Derived Macrophage Cells with M. smegmatis

THP1-cells were cultured in RPMI 1640 medium enriched with 10% FBS, 2mM L-glutamine, 25 mM HEPES, and 1.5 g/L Na_2_CO_3_, kept at 37 °C, 5% CO_2_. Macrophage differentiation was induced by incubating cells with PMA,20 ng/mL for a period of 24 h. Differentiated macrophages were then exposed to *M. smegmatis* at a multiplicity of infection of 20:1 and incubated for 3 h at 37 °C, 5% CO_2_ to allow phagocytosis. After infection, cells were rinsed three times with 1X PBS, followed by resuspension in complete RPMI-containing gentamycin (10 µg/mL) to eliminate any non-internalized bacteria. After three additional 1X PBS washes, the cells were resuspended in fresh medium.

### 4.8. Measurement of Intracellular ROS 

Reactive oxygen species (ROS) production assessed using the DCFDA dye (Merck). Uninfected, vector control *M. smegmatis* (MS_VC) infected, recombinant *M. smegmatis* -expressing *Rv1899c* (MS_*Rv1899c*) infected macrophages were incubated with 10 µM DCFDA for 45 min at 37 °C. Fluorescence intensity was recorded with a microplate reader using excitation at 485 nm and emission at 535 nm and results are expressed as Relative Fluorescence Units (RFU) normalized to cell number.

### 4.9. Intracellular Survival Assays for M. smegmatis

THP1 macrophages were exposed to *M. smegmatis* for infection as described previously. After 3 h, cells were subjected to four consecutive washing with complete RPMI culture medium containing gentamycin (10 µg/mL) to eliminate bacteria outside the cells, and new culture medium was added. Cells were harvested 24 h post-infection. Macrophage monolayers were first washed with PBS before lysis with 0.06% sodium dodecyl sulfate in 7H9 medium, and the lysates were plated on 7H10 agar. Plates were incubated at 37 °C, and the number of viable intracellular bacteria was determined by colony-forming units count after 3–4 days. Experiments were conducted in triplicate.

### 4.10. Total Protein Extraction and Quantification 

Protein extraction was carried out using RIPA buffer (Thermo Fisher Scientific) containing protease and phosphatase inhibitors. Protein levels were determined with the BCA assay (Thermo Fisher Scientific) according to the manufacturer’s protocol, and absorbance was read by 562 nm.

### 4.11. Western Blotting

Protein lysates were prepared from *M. smegmatis*-infected THP-1 macrophages (MOI 20:1) using RIPA buffer (Sigma, USA) supplemented with protease and phosphatase inhibitor cocktails. The lysates were incubated on ice for 30 min and centrifuged at 12,000× *g* for 15 min at 4 °C to remove debris. The supernatant-containing total protein was collected, and the concentration was determined using the Bradford assay (Bio-Rad, USA). Equal amounts of protein (typically 30–40 µg) were resolved on 12% SDS-PAGE gels and electrotransferred onto Immobilon-P PVDF membranes (Bio-Rad, USA). Membranes were blocked with 5% non-fat dry milk in TBST (20 mM Tris-HCl, 150 mM NaCl, 0.1% Tween-20, pH 7.4) for 1 h at room temperature, followed by incubation with specific primary antibodies overnight at 4 °C. The following primary antibodies were used: anti-Beclin-1 (Origin Diagnostics and Research, Kerala, India), anti-ATG5 (Origin Diagnostics and Research, India), anti-CD86 (Cell Signaling Technology, Danvers, MA, USA), anti-LC3 (Origin Diagnostics and Research, India), and anti-β-actin (Origin Diagnostics and Research, India). After washing, membranes were incubated with HRP-conjugated anti-rabbit secondary antibodies for 1 h at room temperature. Protein bands were visualized using enhanced chemiluminescence reagents (ECL, Bio-Rad, USA), and images were captured using a ChemiDoc imaging system (Bio-Rad, USA). Densitometric analysis was performed using ImageJ software (ImageJ 1.54g), and the relative expression of target proteins was normalized to β-actin.

### 4.12. Quantitative Real-Time PCR (qPCR)

The total RNA was extracted from infected and uninfected macrophages using the NucleoSpin RNA extraction kit (Macherey-Nagel, Dueren, Germany) following the manufacturer’s guidelines. In total, 1 µg of RNA was reverse transcribed into cDNA using the Takara PrimeScript 1st strand cDNA synthesis kit. (Takara, Tokyo, Japan). The resulting cDNA was used for qPCR with using TB Green Premix Ex Taq (Takara) on a QuantStudio 5 Real-Time PCR System (Thermo Scientific Applied Biosystems, USA). Relative expression levels were calculated using the 2^−∆∆Ct^, which was employed to calculate comparative expression levels using Beta-actin as the reference.

### 4.13. Cytokine Quantification by ELISA 

Cell culture supernatants were collected at 24h post-treatment. Levels of IL-12p40, TNF-α, and IL-10 were assessed employing ELISA kits (Origin, India), as per the manufacturer’s protocols. Optical density was read at 450 nm using a microplate reader.

### 4.14. Statistical Analysis

The data were expressed as mean values with their standard deviations. The results were analyzed by nonparametric analysis of variance (ANOVA). A *p* value of <0.05 was considered significant.

## 5. Conclusions

In conclusion, this work identifies *Rv1899c* as a pivotal virulence factor determinant in *M. tuberculosis,* crucial for bacterial survival and immune regulation within host cells. Expression of *Rv1899c* in *M. smegmatis* enhanced resistance to acidic and oxidative stress while promoting early intracellular growth by dampening host immune activity. The protein reduced reactive oxygen species levels, suppressed inflammatory cytokines, and inhibited autophagy-related proteins to a decline in M1 macrophage polarization. Inhibition of Rv1899c using 3-aminobenzamide (3-AB) reduced intracellular bacterial survival and restored IL-12B expression, effects that were further strengthened when combined with the HDAC inhibitor C1994. Structural and computational analyses confirmed the stability and high stereochemical quality of the Rv1899c macrodomain, while dynamic simulations supported strong and stable interactions with 3-AB, driven mainly by van der Waals and electrostatic forces. Overall, these results reveal *Rv1899c* as an important mediator of stress adaptation and immune suppression, highlighting its promise as a potential target for tuberculosis therapy.

## Data Availability

The original contributions presented in this study are included in the article. Further inquiries can be directed to the corresponding author.
